# How do women want to receive information about non‐invasive prenatal testing? Evidence from a discrete choice experiment

**DOI:** 10.1002/pd.6243

**Published:** 2022-10-02

**Authors:** Stuart J. Wright, Garima Dalal, Caroline M. Vass, Susanne Georgsson, Katherine Payne

**Affiliations:** ^1^ Manchester Centre for Health Economics The University of Manchester Manchester UK; ^2^ RTI Health Solutions Manchester UK; ^3^ Red Cross University College of Nursing Stockholm Sweden

## Abstract

**Objective:**

Non‐invasive prenatal testing (NIPT) identifies the risk of abnormalities in pregnancy, potentially reducing the risk of miscarriage associated with invasive tests. This study aimed to understand the preferences of current and future mothers about the content, format and timing of information provision about NIPT.

**Methods:**

An online discrete choice experiment was designed comprising four attributes: when in the pregnancy information is provided (4 levels); degree of detail (2 levels); information format (6 levels); cost to women for gathering information (5 levels). Respondents included women identified by an online‐panel company in Sweden. The mathematical design was informed by D‐efficient criteria. Choice data were analysed using uncorrelated random parameters logit and latent class models.

**Results:**

One thousand Swedish women (56% current mothers) aged 18–45 years completed the survey. On average, women preferred extensive information provided at/before 9 weeks of pregnancy. There was heterogeneity in preferences about the desired format of information provision (website, mobile app or individual discussion with a midwife) in the population.

**Conclusion:**

Women had clear preferences about the desired content, format and timing of information provision about NIPT. It is important to tailor information provision to enable informed choices about NIPT.

## Key points

What's already known about the topic?

- Non‐invasive prenatal testing (NIPT) is an emerging approach that detects fetal DNA from a maternal blood sample to identify the risk of abnormalities in pregnancy.
- Parents must be given the correct type and amount of information in an appropriate format. The information is needed to provide consent for undergoing NIPT.

What does the study add?

- A discrete choice experiment was used to quantify the preferences of current and future mothers for content, format and timing of the provision of information about NIPT.
- Women had a strong preference for extensive information provided early in pregnancy using a variety of formats (text‐based websites; mobile apps; individual discussions with a midwife).
- Substantial heterogeneity in preferences was identified suggesting a tailored approach to effectively provide information about NIPT is required.

## 1 INTRODUCTION

Non‐invasive prenatal testing (NIPT) is a prenatal testing approach that detects circulating fetal DNA from a maternal blood sample.^1^ The suggested advantages of NIPT are its potential to reduce the risk of miscarriage by negating the need for invasive tests and the opportunity to screen women early in pregnancy.^2^ The suggested disadvantages of NIPT are that it cannot detect non‐genetic abnormalities such as neural tube defects^3^ and is less accurate in certain groups who may still require an invasive diagnostic test for a confirmatory result.

This study focuses on the public provision of NIPT as it is offered free of charge to women in some regions of Sweden whose first trimester combined ultrasound and blood (CUB) test shows an increased probability of chromosomal abnormalities 13, 18 or 21, those who have previously been expecting a child with these chromosomal abnormalities and those whose previous ultrasounds or other tests have shown abnormalities.^4^ The availability of the test varies across regions in Sweden and in the regions where it is available, NIPT is offered between weeks 12–14 of the pregnancy after the CUB test.^5^ Due to this variation, there are no standardised materials for providing women with information about the test but some regions have developed materials that are used in all antenatal units within the region. Additionally, information related to NIPT is available on a regularly updated webpage which covers Sweden as a whole and the each of its regions.^6^

The introduction of NIPT into prenatal screening creates a change to the decision faced by women deciding whether to take the test, who must now balance the increased safety with increased waiting times and the risk of a false result. Although pre‐test counselling and information provision is recommended in published guidelines,^7^ a survey of American women who experienced NIPT found that many women did not fully appreciate the potential disadvantages of the test despite reporting satisfaction with their understanding of NIPT and the process.^3^ Discrete choice experiments (DCEs) are a commonly used stated preference method of eliciting and quantifying individuals' preferences about the benefits and risks associated with new interventions.^8^, ^9^, ^10^ In a DCE, participants are asked to select their preferred alternative in a series of hypothetical choice scenarios. Fifteen studies eliciting women's preferences for prenatal testing using a DCE have been published.^11^ However, existing DCEs only assessed which test characteristics influence women's preferences, did not specifically evaluate how women would like to receive information about the test and have mostly been conducted in the UK. Effective communication of information about NIPT is important to enable women to make an informed decision. There is evidence that informed decision‐making may help reduce parents' anxiety and improve their feelings of autonomy or control over their pregnancy.^12^ There is qualitative and quantitative evidence to suggest that NIPT is generally preferred to invasive procedures with women preferring information about as many conditions as possible (see^13^, ^14^, ^15^). There is a paucity of evidence about how women want to receive information about NIPT and few studies have been conducted in Sweden. An international DCE study quantified preferences of a sample of women from different countries (including Sweden) for factors that affect their decision‐making around prenatal genomic tests.^16^ However, this study assessed preferences for testing, not information provision. Understanding how to effectively communicate the information to expectant mothers may mean policy makers can provide details in a way that maximises benefit to the mother and baby alike. The content, format and timing of information provision is likely to influence how ‘prepared’ women feel about making the decision to participate in newborn screening and testing programmes.^12^ This study aimed to understand the preferences of current and future mothers about the content, format and timing of information provision about NIPT.

## 2 MATERIALS AND METHODS

An online survey comprising a DCE with additional supplementary questions was used to quantify the preferences of a sample of women living in Sweden. Data were collected between August and September 2019. The DCE was designed, analysed and reported in line with published guidelines.^17^, ^18^ Ethical approval was obtained from The Swedish Regional Ethics Board (reference: DNR 2019‐04122).

### 2.1 Survey design

The survey was programmed using HTML for online administration using SSI Web 8.3.8 Sawtooth software.^19^ It was designed in English, forward translated to Swedish by an academic native Swedish researcher (SG) and then backward translated by Wolfestone Translation Ltd.^20^ and an independent academic native Swedish researcher. The final survey was presented to respondents in Swedish. The final version of the survey (see Supplementary Appendices S1 and S2) comprised three sections: study background describing NIPT; the choice questions; and questions asking the respondents about themselves.

### 2.2 DCE design

The DCE was framed to ask respondents to make a choice between two unlabelled alternatives of receiving NIPT information (options A and B) and an opt‐out alternative representing ‘no information’. Including an opt‐out alternative allows the analysis to estimate the probability of women choosing to have ‘no information’.

**FIGURE 1:**
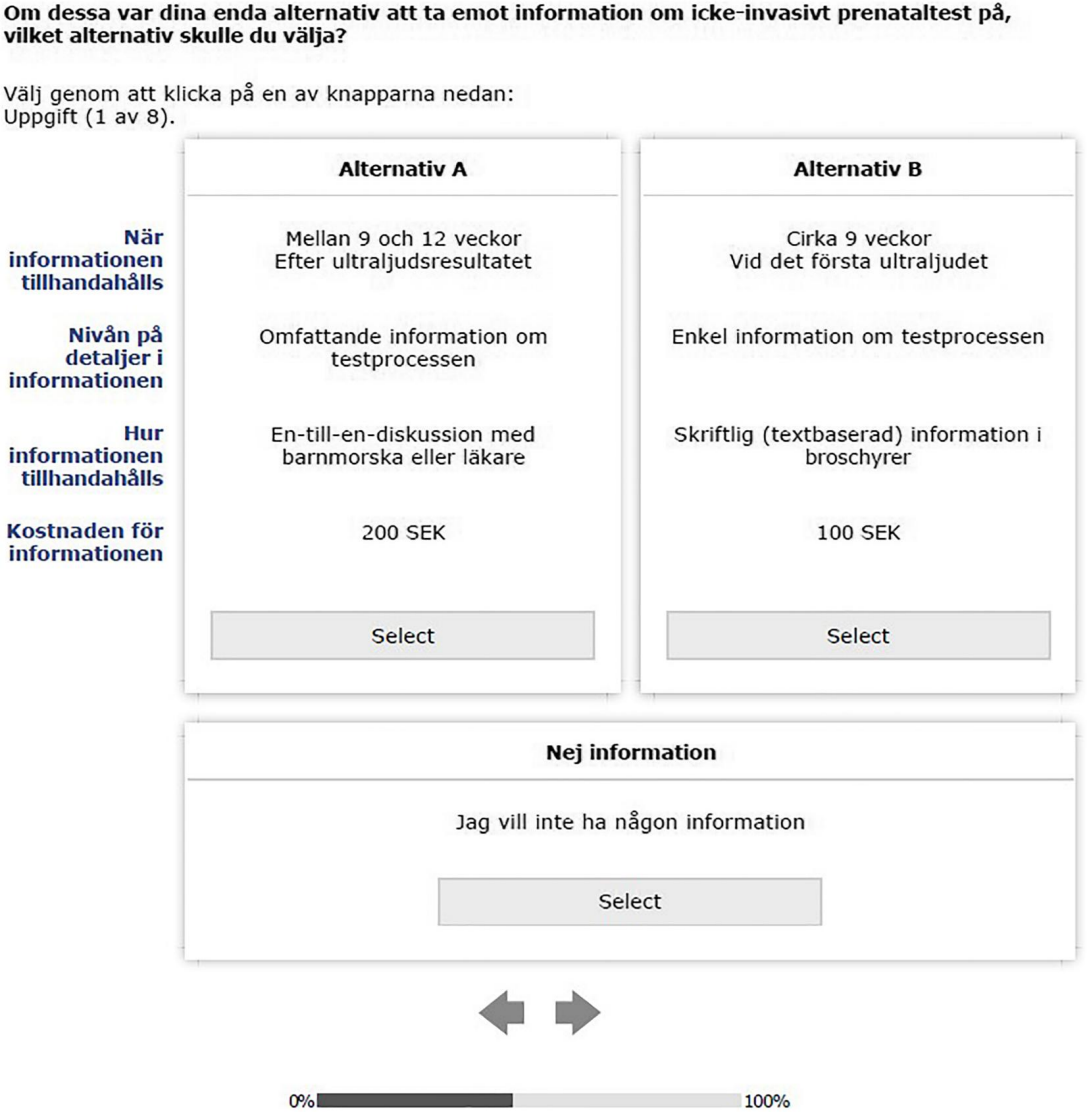
An example of a choice set used in the survey (Swedish)

**FIGURE 2:**
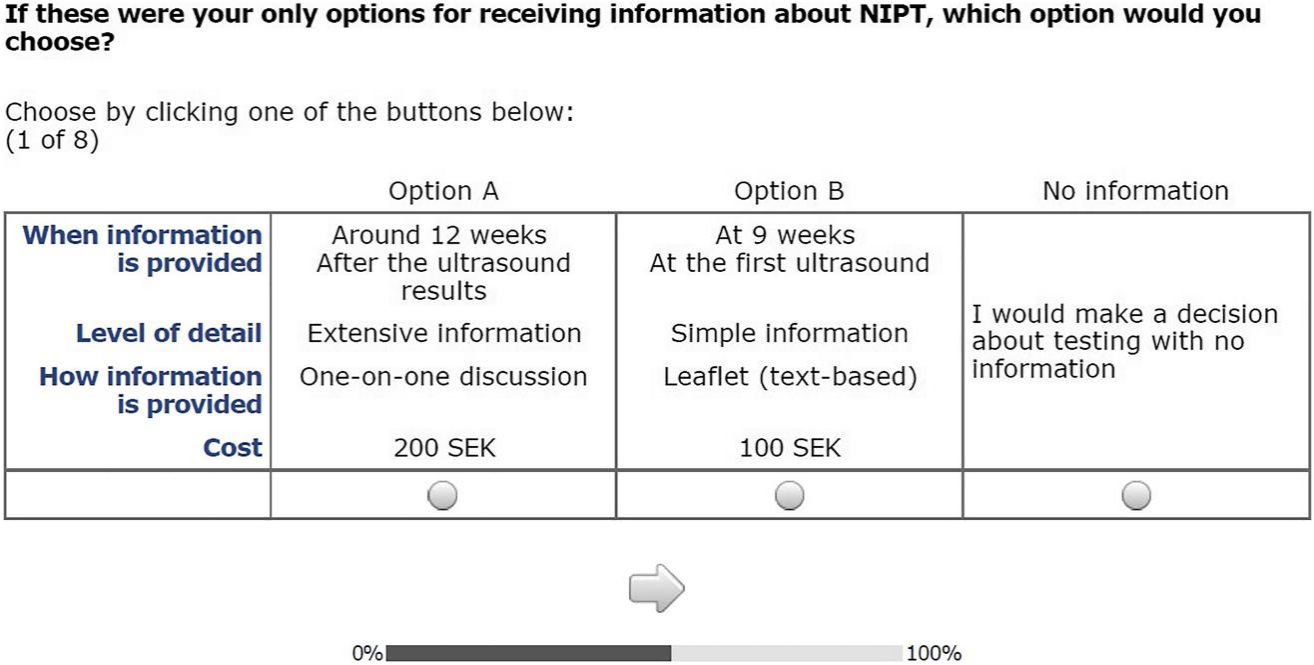
An example of a choice set used in the survey (English)

Table 1 describes the four attributes and relevant levels that were chosen to address the choice question: ‘If these were your only options for receiving non‐invasive prenatal test information, which option would you choose?’ Figures 1 and 2 show an example choice question in Swedish and English. All attributes were generic, rather than specific to each alternative. Attribute and level selection used a mixed methods approach including a literature review^11^ and input from two experts: health psychologist working in newborn screening (with 20 years of experience) and a nurse midwife (with 30 years of experience).

**TABLE 1:** Attributes and levels used in the discrete choice experiment

Attributes	Rationale for the attribute	Levels	Rationale for the levels
When the information is provided^a^	NIPT can be conducted up until childbirth. It is unclear when the women want information about the testing relative to the key milestones in pregnancy.	At first confirmation of pregnancy (<9 weeks) At combined test (∼9 weeks) After combined test results (9–12 weeks) Later in pregnancy (12–18 weeks)	The levels represented key milestones in pregnancy. The inclusion of the level ‘later in pregnancy’ reflects that some women may not make termination decisions and therefore want information later, just so they ‘know’.
Level of detail in the information^a^	It is unclear how much detail women want about NIPT and the process.	Extensive information: Giving detailed description about the testing process including a description about the procedure such as how the sample is taken, how it is then analysed and the quality of the analysis (test accuracy) and information about the conditions that are being screened for. Simple information: Giving a brief description about the testing process including a brief description about how the sample is taken.	The levels were chosen to understand whether women prefer to receive extensive information or minimal information so they can do further research themselves.
How the information is provided^a^	It is unclear what format of information communication women want.	A one‐on‐one discussion with a midwife or doctor At an antenatal group discussion Written (text‐based) leaflets Text‐based information via a website App Interactive website	The levels represented different modes of information provision. Antenatal care in Sweden is based on visits to a midwife during pregnancy but other potential modes of information provision exist which may be preferred by pregnant women. These were informed by an existing discrete choice experiment in newborn bloodspot screening (NBS). In this NBS study, the levels were based on how information is currently provided in the UK. The level ‘interactive website’ was added in this study to understand the potential demand for this mode of information provision.
The cost of the information (out‐of‐pocket costs incurred)^b^	Although NIPT is free to women who are deemed high‐risk after the first trimester combined ultrasound and biochemical (CUB) screening test, they will still incur out‐of‐pocket costs because of the need to travel to the hospital and take time away from other activities.	50 SEK 100 SEK 200 SEK 1000 SEK 2000 SEK	The levels represented the cost that women may incur as a result of seeking information about NIPT. For example, travel costs, time taken off work, food etc. The cost of NIPT is not included in these levels as it is offered free of charge to high‐risk women in some regions of Sweden. These levels were adapted from the NBS discrete choice experiment.

^a^ This variable was effects coded in the data.

^b^ This variable was coded as continuous in the data.

### 2.3 Training materials

Training materials were used at the start of the survey to ‘set the scene’ and explain key concepts to respondents. An interactive Prezi presentation was created to use as training materials and inserted at the start of the survey (see Supplementary Appendix S3).^21^ It covered: what is NIPT; why is NIPT offered to pregnant women; other different types of tests available; and how information about antenatal screening could be provided. The required content of the training materials was informed by contacting 50 antenatal care units in Sweden and requesting the written information that they offer to women (if offered). Responses were received from 38 units which suggested that information is provided in varied formats across Sweden as some clinics only provide information verbally, some only provide written information and others provide information in both formats. Responses to the survey also indicated that verbal information is provided using different modes of communication such as during one‐to‐one discussions or in a group with other expectant mothers.

### 2.4 Experimental design

The experimental design informs the specific combinations of attributes and levels that respondents evaluate in the choice questions and number of choice questions.^22^ A complete factorial experimental design would result in 28,680 possible profile combinations (see Table 1). To reduce the number of choice sets to a manageable size and to allow the researchers to prevent illogical combinations occurring, a D‐efficient design using the modified Federov algorithm was created using Ngene software.^23^ This approach selected a subset of total possible choice sets (*n* = 32) by generating a mathematical design while minimising the D‐error. In line with published evidence supporting the maximum number of choice sets one respondent can complete,^24^ each respondent was asked to complete eight choice sets. This resulted in four surveys, each comprising eight choice sets, to achieve the required 32 choice sets. The surveys were created by allocating the 32 choice sets to one of four blocks while maintaining a balance in terms of statistical, design and response efficiencies. An internal validity check question was not included in the experimental design because three of the four attributes were categorical in nature meaning it was not possible to assign a dominant preference in the design of a choice set.^25^

### 2.5 Background questions

The online survey included background questions to provide potential predictors of preferences within the sample. These questions used a mixture of discrete, Likert‐rating and open‐ended questions. Respondents were asked about socio‐demographic status (e.g., age, education, employment status); experiences of pregnancy and screening (e.g., have you had any pregnancies?, have you ever had a genetic test? *etc*); degree to which a person engages with information provision or is apprehensive about using health information (using the Health Information Orientation Scale^26^, ^27^); and current health (using EQ‐5D‐5L^28^).

### 2.6 Piloting

A pre‐pilot study used think‐aloud face‐to‐face interviews with three women from The University of Manchester. This process identified adjustments needed to the wording of the survey to improve framing of the questions. A quantitative pilot with a sample of 50 Swedish women was used to test the online survey, with additional free‐text boxes, to allow respondents to offer suggestions about improvements and identify any technical issues with the online survey. The pilot study sample was recruited using an online panel provider (Dynata; formerly Research Now).^29^ This pilot identified that the highest level for the cost attribute (which denotes cost to women for gathering information, not cost of NIPT) needed to be larger and this was subsequently doubled. A second quantitative pilot with a different sample of 50 Swedish women was then conducted. No changes were made after this second pilot study.

### 2.7 Study population and sample

The relevant study population was (current or future) mothers who might be in a position to make the decision about NIPT. This was because it changes the risk of procedure related to injury to the mother and/or child and the decision and results are ultimately the mother's choice.

A sample of women, currently with and without children, between the ages of 18 and 45 years, living in Sweden were recruited by Dynata. The target sample size of 1000 was chosen to allow analysis of the preferences of different subgroups. The amount of compensation received by the respondents completing the survey through the online panel provider was in line with Dynata policies and was not determined by the University of Manchester researchers. The survey link shared by the panel provider with participants included an anonymised ID number which was recorded by the researchers and passed back to the panel provider when a participant completed the survey in order for them to be reimbursed.

Potential respondents were sent a link to the online survey and no reminders were used. The start of the survey contained two screening questions to remove men and also women aged 46 years or older.

### 2.8 Data analysis

An analysis plan was specified that respondents who did not complete the whole survey will be excluded. Stata 14 was used for all analyses.^30^ Descriptive statistics were produced for respondents included in the final sample for analysis. Tests were conducted to determine whether respondents had always chosen the profile with higher or lower cost or extensive or simple information.

Choice data were analysed using regression‐based econometric methods using two steps. Step one used a conditional logistic regression model (CLM) to estimate the aggregate preferences of the whole sample completing the survey. A series of analysis were then run (see Supplementary Appendix S4) to identify the correct model specification and functional form for the parameters included in the regression model. This process recorded the log‐likelihood, Akaike Information Criterion (AIC),^31^ and Bayesian Information Criterion (BIC)^32^ to inform model specification and functional form. This process identified that an uncorrelated random parameters logit (RPL) model^33^ with effects‐coded variables for each attribute except cost, which was coded as continuous, was the optimal regression model.

Step two used a latent class analysis (LCA) regression model to characterise potential sources of heterogeneity in the preferences in the sample of women. LCA identifies groups (classes) of respondents within the sample who have similar preferences. The final stage in LCA then involves adding demographic factors to the LCA regression model to predict which types of people tended to belong to each of the identified class. The optimum number of classes and membership of each class was identified using log‐likelihood, AIC, BIC and the size of the standard errors of the estimated coefficients (aiming to minimise the size of the standard errors).^34^

### 2.9 Balancing benefits and harms

The outputs of the uncorrelated RPL analysis can be used to calculate the balance of benefits and harms for the included attributes in the DCE. This ratio of benefits: harms represents the amount of one attribute a respondent is willing to give up for a unit change in another and is generated by calculating the marginal rate of substitution (MRS). The MRS is calculated by dividing the estimated coefficient of interest by an attribute used as a ‘common’ denominator (sometimes called a ‘value attribute’). Using the cost attribute as the denominator means that the willingness‐to‐pay (WTP) for the format, timing and level of detail of information can be calculated.

## 3 RESULTS

The final sample comprised 1000 women (see Table 2) that completed the survey. The median time taken to complete the survey was 15 min. Just over half the sample was aged between 18 and 35 years old and reported being currently in good health (see Supplementary Appendix S5). Just over two‐fifths (44%) of the sample reported having no children and were currently not pregnant and had no previous pregnancies. Just over 20% of the women had ever been offered a prenatal test and of these, 16% had ever accepted a prenatal test.

**TABLE 2:** Sample characteristics

Characteristics	*n* = 1000
Age band	
18–35 years	560 (56%)
36–45 years	440 (44%)
Number of children	
None	440 (44%)
One	238 (23.8%)
Two	205 (20.5%)
Three	117 (11.7%)
Currently pregnant	
Yes	86 (8.6%)
No	896 (89.6%)
Don't know	18 (1.8%)
Previous pregnancies	
None	384 (38.4%)
One	228 (22.8%)
Two	199 (19.9%)
Three or more	189 (18.9%)
Marital status	
Single	257 (25.7%)
Separated	54 (5.4%)
Live with partner	341 (34.1%)
Married	303 (30.3%)
Widow	1 (0.1%)
Other	44 (4.4%)
Religion	
None	568 (56.8%)
Christian	322 (32.2%)
Buddhist	12 (1.2%)
Jewish	4 (0.4%)
Hindu	6 (0.6%)
Muslim	70 (7%)
Other	17 (1.7%)
Highest education level	
No formal qualifications	2 (0.2%)
Elementary or primary school	59 (5.9%)
Two years of high school	52 (5.2%)
Three to four years high school	332 (33.2%)
Folk high school or similar	51 (5.1%)
University or college <3 years	110 (11%)
University or college 3 years or longer	336 (33.6%)
Doctorate	14 (1.4%)
Other higher/secondary education	42 (4.2%)
Occupation	
Full‐time employee	441 (44.1%)
Part‐time employee	133 (13.3%)
Self‐employed	36 (3.6%)
Unemployed	73 (7.3%)
Retired	15 (1.5%)
At home with children/home care	57 (5.7%)
Student	153 (15.3%)
Freelance	10 (1%)
Long‐term sick	53 (5.3%)
Temporarily unemployed	28 (2.8%)
Ever been offered a prenatal test	
Yes	218 (21.8%)
No	734 (73.4%)
Don't know	48 (4.8%)
Ever accepted a prenatal test	
Yes	162 (16.2%)
No	785 (78.5%)
Don't know	53 (5.3%)
Genetic illness in immediate family	
Yes	263 (26.3%)
No	654 (65.4%)
Don't know	83 (8.3%)
Perceived risk of Down's syndrome	
High	14 (1.4%)
Quite high	66 (6.6%)
Average	227 (22.7%)
Low	441 (44.1%)
Don't know	252 (25.2%)
Mean information engagement score (measured on a scale of 0–4)	2.74
Mean information apprehension score (measured on a scale of 0–4)	1.89

*Note*: Data are presented as *n* (%).

Respondents indicated an overall good level of engagement with information provision given a mean score of 2.74 on a predefined scale that ranged from zero (no engagement) to four (high engagement). Respondents also demonstrated a relatively low level of information apprehension with a mean score of 1.89 on a scale from zero (no apprehension) to four (high apprehension).

Few respondents always made their choices based on a single attribute. Only 28 (2.8%) of the 1000 respondents always chose the NIPT information with the lower cost, while four (0.4%) always chose the profile with the higher cost. Only 15 (1.5%) respondents always chose to receive more detailed information while only five (0.5%) always chose to receive simple information.

### 3.1 Women's preferences

Table 3 presents the results of the uncorrelated RPL model with the cost attribute specified using the piecewise functional form (see Supplementary Appendix S4). The signs of all the estimated coefficients were in line with a priori expectations regarding the direction of the effect of an attribute level on women's preferences. The positive and statistically significant alternative‐specific constant (ASC) term suggested that women place a high intrinsic value on receiving some form of information compared with no information. The associated WTP for information compared with no information was 7861 SEK.

**TABLE 3:** Results from the uncorrelated random parameters logit model

Attribute/Level	Coefficient	Coefficient lower 95% CI	Coefficient higher 95% CI	WTP (SEK)^a^	*p*‐Value
Constant: Value of a NIPT information package with the average effect for when information is provided, level of detail and mode compared to receiving no information	3.505***	3.055	3.956	7,861	0.000
Information before 9 weeks	0.454***	0.215	0.693	9,941	0.000
Information at 9 weeks	0.521***	0.355	0.686	10,247	0.000
Information between 9 and 12 weeks	0.088	−0.061	0.236	8,262	0.247
Information after 12 weeks	−1.062***	−1.312	−0.812	2,993	0.000
Extensive information	0.499***	0.332	0.665	10,147	0.000
Simple information	−0.499***	−0.665	−0.332	5,575	0.000
Leaflet‐based information	0.255	−0.010	0.520	9,029	0.060
Text‐based information via website	0.406***	0.221	0.590	9,720	0.000
App‐based information	0.250**	0.083	0.418	9,008	0.003
Interactive website‐based information	−0.085	−0.239	0.069	7,471	0.278
Information in discussion with midwife	0.319***	0.147	0.491	9,322	0.000
Information in group discussion	−1.145***	−1.402	−0.887	2,616	0.000
Cost under 1000 SEK^a^	−0.002***	−0.002	−0.002		0.000
Cost over 1000 SEK	0.000	0.000	0.000		0.089
Number of observations	24,000				

Abbreviations: CI, confidence interval; NIPT, non‐invasive prenatal testing; WTP, willingness‐to‐pay.

^a^ As the effect of cost is non‐linear, WTP values were calculated for a programme of information (the constant and a change in the specified level) compared to no information as opposed to just a change in the level. For example, the WTP for information at 9 weeks (10,247 SEK) represents the value of a programme of information provision where information is provided at 9 weeks, with the mean effect for level of detail, mode of information and 0 cost compared to receiving no information.

*p < 0.05; **p < 0.01; ***p < 0.001.

Women preferred information before or at 9 weeks of pregnancy and were averse to information after 12 weeks. Extensive information was preferred to simple information and women wanted to receive information via a text‐based website, a discussion with their midwife, or through an app but not in a group discussion.

All attribute level coefficients were statistically significant predictors of choice at the 5% level except information provided between 9 and 12 weeks, via an interactive website or leaflet and information that cost women over 1000 SEK in out‐of‐pocket costs. This indicated that respondents considered most information provided to them when making a choice. Women expressed the largest WTP for information being provided at 9 weeks of pregnancy, followed by extensive information being provided (10,247 SEK and 10,147 SEK, respectively).

### 3.2 Heterogeneity in women's preferences

The LCA identified three classes as the optimal model (see Table 4). Nearly half (48%) of the sample were predicted to belong in class 1. Women in this class preferred extensive information in a leaflet or via a text‐based website before 12 weeks. The size of the ASC term suggested that women in this class placed a relatively high intrinsic value on information about NIPT but were also quite sensitive to the cost of gathering information. Women in this class wanted information via text‐based websites but not via an interactive website or in a group discussion and were more likely to agree with the statement ‘I believe that terminations should be allowed for all different reasons’ (see predictors of class membership in Table 5). Women in class 1 expressed the greatest WTP of 1621 SEK for information provided via text‐based websites with a mean effect for level of detail, when the information is provided and a cost of 0 SEK when compared with no information.

**TABLE 4:** Results of the latent class analysis

Attributes/Levels	Class 1 (48%)^a^			Class 2 (34%)^a^			Class 3 (18%)^a^		
	Coefficients	*p*‐Value	WTP (SEK)^b^	Coefficients	*p*‐Value	WTP (SEK)^b^	Coefficients	*p*‐Value	WTP (SEK)^b^
Constant: Value of NIPT information with mean effect for time, level of detail and mode compared to no information	2.586***	0.000	994	3.264***	0.000	11,214	−2.460***	0.000	−1,757
Information before 9 weeks	0.518*	0.012	1,336	0.437*	0.015	12,671	1.631***	0.000	−592
Information at 9 weeks	0.414**	0.001	1,266	−0.217	0.097	10,491	0.831***	0.000	−1,163
Information between 9 and 12 weeks	0.358**	0.004	1,229	−0.381*	0.011	9,943	0.082	0.317	−1,699
Information after 12 weeks	−1.290***	0.000	498	0.161	0.204	11,751	−2.544***	0.000	−3,574
Extensive information	0.608***	0.000	1,396	0.021	0.419	11,284	1.133***	0.000	−948
Simple information	−0.608***	0.000	760	−0.021	0.419	11,144	−1.133***	0.000	−2,567
Leaflet‐based information	0.440*	0.020	1,284	0.050	0.399	11,382	1.422***	0.000	−741
Text‐based information via website	0.946***	0.000	1,621	−0.329**	0.003	10,116	1.170***	0.000	−921
App‐based information	0.119	0.205	1,070	−0.134	0.181	10,767	0.736***	0.000	−1,231
Interactive website‐based information	−0.577***	0.000	772	0.044	0.359	11,360	−0.381*	0.029	−2,029
Information in discussion with midwife	−0.043	0.602	978	0.448***	0.000	12,708	−0.747**	0.003	−2,291
Information in group discussion	−0.885***	0.000	654	−0.079	0.324	10,951	−2.201***	0.000	−3,329
Cost under 1000 SEK	−0.003***	0.000		0.000	0.076		−0.001**	0.003	
Cost over 1000 SEK	−0.002***	0.000		0.000*	0.031		0.001	0.173	
Sample size across 3 classes	1,000								

Abbreviations: NIPT, non‐invasive prenatal testing; WTP, willingness‐to‐pay.

^a^ Each respondent is assigned a probability of being in a certain class as the class membership cannot be established with certainty.

^b^ As the effect of cost is non‐linear, WTP values were calculated for a programme of information (the constant and a change in the specified level) compared to no information as opposed to just a change in the level. For example, the WTP for information at 9 weeks in class 1 (1,266 SEK) represents the value of a programme of information provision where information is provided at 9 weeks, with the mean effect for level of detail, mode of information and 0 cost compared to receiving no information.

*p < 0.05; **p < 0.01; ***p < 0.001.

**TABLE 5:** Predictors of class membership from latent class analysis

Predictors of classes	Class 1		Class 2		Class 3	
	Coefficient	*p*‐Value	Coefficient	*p*‐Value	Coefficient	*p*‐Value
Constant	0.300		1.260		−1.560	
How easy was it to choose	−0.045	0.153	−0.169***	0.000	0.214***	0.000
Self‐perceived low risk of having child with Down's syndrome	−0.026	0.312	−0.255***	0.000	0.281***	0.000
Agreement with statement ‘termination should be allowed for all different reasons’	0.118**	0.004	0.107*	0.013	−0.226***	0.000

*p < 0.05; **p < 0.01; ***p < 0.001.

Around one‐third (34%) of the sample were predicted to belong to class 2. Women in this class had a high intrinsic value for information and lower sensitivity to cost compared with those in class 1, resulting in higher WTP values. These women wanted information before 9 weeks and valued information provided in discussions with their midwife. They did not want information to be provided via a text‐based website. Women in this class were more likely to state that they believed they were at higher risk of having a child with Down's syndrome, were more likely to say they found the choice tasks difficult and were more likely to agree with the statement ‘I believe that terminations should be allowed for all different reasons’ (see Table 5). Women in class 2 expressed the largest WTP of 12,708 SEK for information provided via individual discussions with a midwife.

Just under one‐fifth (18%) of the sample were predicted to belong to class 3. Women in this class had a low intrinsic value for information, generally preferring to receive no information. When they did choose to receive information, they had similar preferences for how that information was delivered as the women in class 1. Women in class 3 were more likely to report that they believed their risk of having a child with Down's syndrome was low, more likely to state that completing the choice tasks was easy and more likely to disagree with the statement ‘I believe that terminations should be allowed for all different reasons’ (see Table 5).

## 4 DISCUSSION

This study quantified the preferences of a sample of women, representing current and future mothers, living in Sweden for information provision related to NIPT. The study also identified the determinants of variation in the preferences within the sample of women. The results provide evidence that, on average, women in Sweden would like information when deciding whether to participate in NIPT. The sample demonstrated a strong preference for receiving NIPT‐related information before or at 9 weeks of pregnancy and for receiving extensive information, via a text‐based website, individual discussions with a midwife or a mobile app. Cost was a statistically significant predictor of choice indicating that higher out‐of‐pocket costs associated with obtaining information about NIPT negatively influenced women's choices. However, the impact of this attribute on preferences was relatively small when compared with the influence of the other three attributes (when the information is provided; how the information is provided; level of detail contained in the information).

The presence of subgroups of women with specific preferences about information provision was identified. Examining the preferences of these subgroups uncovered three distinct groups of women who prefer to receive information about NIPT in different ways. The predominant subgroup represented women who were cost‐sensitive and would like information but potentially only as a useful addition to their maternity care. Around a third of the sample formed another subgroup, who perceived their risk of having a child with Down's syndrome to be high and placed a greater intrinsic value on information provision. A third subgroup comprised women who were more likely to be against termination, perceived themselves to be at low risk of having a baby with Down's syndrome, or found the decision of whether to participate in NIPT easy to make. This subgroup of women did not want information about NIPT but would rather read extensively about NIPT in their own time. They also did not value pre‐scheduled face‐to‐face group discussions or individual discussions with a midwife. One reason for this could be that women in this subgroup would like to have the information to hand when discussing NIPT with friends or family.^35^, ^36^, ^37^ There is some evidence to support that some women are proactive in their healthcare decision‐making and prefer to gather information from a range of sources (such as leaflets and websites) in addition to appointments with healthcare professionals.^38^

The preferences identified in this sample of women indicate that although some women value discussions with a midwife, this is not the case for all; some would prefer to receive information through other sources. This echoes findings from previous studies conducted in Sweden which found that pregnant women and high school students would prefer to receive or seek information about prenatal testing on the Internet, through written materials or a combination of oral and written communication.^39^, ^40^ The preference for receiving information through midwives and a variety of other sources has also been reported for other countries such as the UK and the US.^35^, ^36^, ^38^, ^41^ Similarly, studies have previously found that women prefer to receive information early in the pregnancy.^37^, ^42^

While many DCEs have been conducted in the areas of screening and diagnosis, few have investigated preferences for information to inform patients' decision‐making around these interventions. The finding that women prefer to receive information through individual discussions, and that they prefer extensive information, has previously been reported for a newborn bloodspot screening programme in the UK.^43^ However, the women in the present study showed a greater preference for text‐based information available on the Internet or in a leaflet than in the newborn bloodspot screening study.

The findings from this study may be generalisable to other countries with similar healthcare systems. For example, NIPT provision is broadly similar in some European countries^44^ where the test is covered by public healthcare system for women deemed to be at high risk for chromosomal anomalies. However, in other countries, the test is self‐financed or there is a variation in how the test is offered (publicly or privately). In countries with a low uptake of NIPT, it is important to understand how information should be provided to women who are considering whether to provide consent for NIPT.

There are some limitations associated with this study. Although the sample size of 1000 women may be considered large, the results may not be generalisable to the wider population of women in the position of deciding whether to receive information about NIPT. Our study also had a limited mix of ethnicities which may influence the generalisability of the findings. Future work could explore the preferences of different ethnic groups and cultures as they may have a different set of beliefs which may influence the decision of whether, when and how to seek information about NIPT.

A key strength of this study was the use of a questionnaire survey of antenatal care units in Sweden to inform training materials and attribute development. Training materials are an important part of a DCE survey as they provide an introduction to the clinical area and help the respondent understand the context of the choice questions. When designing a DCE survey, it is also important to ensure that the attributes and attribute levels are realistic and based on what would occur in practice. The relatively large sample size completing the survey made it possible to explore the data, assess the validity of the survey, and identify subgroups of women who may have different preferences. The trading behaviour aligned with a priori expectations and only a few women (∼0.4%) preferred higher cost alternatives, suggesting the survey was generally well understood by respondents. The WTP values enable a monetary valuation of different aspects of NIPT information provision. These can be useful for comparing across different classes or subgroups of women (as shown here) but also to understand the potential demand of a healthcare programme with certain characteristics. Specifically, the WTP values could be used in a cost‐benefit analysis to understand the feasibility and acceptability of a mobile app to provide NIPT information.

Future work is needed to understand the economic impact of different service models of information provision related to NIPT testing in Sweden. A previous systematic review of economic evaluations of NIPT suggested that the impact of information provision, in terms of cost and the effect on parents' wellbeing, is rarely accounted for in such studies.^45^ Similar findings have also been reported for the provision of information about newborn bloodspot screening.^46^ Given the number of women potentially eligible for neonatal screening, changes in the resource requirements for providing information, for example, due to the introduction of a more complex test such as NIPT, could result in significant additional costs for the health system. In addition, poorly designed or provided information may fail to prepare parents for the outcomes of testing leading to an impact on their health‐related quality of life. As such, economic evaluations of NIPT which fail to account for wider changes in the informational requirements of parents may provide misleading estimates of the cost‐effectiveness of the intervention.

## 5 CONCLUSION

The findings from this study showed that women living in Sweden have a strong preference for receiving extensive information about NIPT via individual discussions with a midwife, text‐based websites and mobile apps before or at 9 weeks of pregnancy. Importantly, this study identified there were subgroups of women with specific preferences about information provision can be used to design tailored leaflets, text‐based websites and mobile apps about NIPT.

## CONFLICT OF INTEREST

The authors have no conflict of interest to declare.

## ETHICS STATEMENT

Ethical approval (reference: DNR 2019‐04122) for this study was granted by the Swedish Regional Ethics Board.

## Supporting information

Supporting Information S1

## Data Availability

The survey used to collate the data for this study is made available in the supplementary appendice as a pdf version of the online survey. Data sharing is not appropriate for this article as analysis of the dataset generated is on‐going.
