# Supplementary material for: How do women want to receive information about non‐invasive prenatal testing? Evidence from a discrete choice experiment
Source: Prenat Diagn. 2022 Oct 2;42(11):1377–89. doi: 10.1002/pd.6243 (PMC9828485; doi:10.1002/pd.6243)
Supplement: Supplementary file 1 — Supporting Information S1 [file PD-42-1377-s001.docx]

**List of Supplementary appendices for:** Quantifying Mothers’ Preferences for Providing Information about Non-Invasive Prenatal Testing in Sweden: Evidence from a Discrete Choice Experiment by *Stuart J Wright, Garima Dalal, Caroline M Vass, Susanne Georgsson, Katherine Payne.*

*Corresponding author*: Katherine Payne: [katherine.payne@manchester.ac.uk](mailto:katherine.payne@manchester.ac.uk)

Supplementary Appendix S1: The survey in Swedish

Supplementary Appendix S2: The survey in English

Supplementary Appendix S3: Prezi presentation used for training materials

Supplementary Appendix S4: Selection of functional form of the model

Supplementary Appendix S5: Illustration of reported health status in survey respondents

**Supplementary Appendix S1 – The survey in Swedish (see pdf)**

**Supplementary Appendix S2 – The survey in English (see pdf)**

**Supplementary Appendix S3: Prezi presentation used for training materials**

For the interactive presentation used as training materials, please use DOI: 10.5281/zenodo.6840640 or click [here](https://doi.org/10.5281/zenodo.6840641).

**Supplementary Appendix S4 – Selection of functional form of the model**

A series of models using different functional forms were estimated to determine:

1. The appropriate number of alternative-specific constants to include in the regression model
2. The presence of non-linearity in preferences for the cost attribute
3. The best fitting model for the observed choice data

Test 1: Number of alternative-specific constants specified

*Objective of test 1:* To determine whether one opt-in constant (representing information compared with no information) or two separate opt-in constants (representing each of the two scenarios in the choice-set) was a more appropriate model specification.

*Methods for test 1:* Two separate conditional logit models (CLM) were run for two specifications of the alternative-specific constant:

Option 1: Model with one constant

Option 2: Model with two constants

Both models assumed a linear and continuous specification for all attributes. The CLM model with the lower Bayesian Information Criterion (BIC) was chosen as the more appropriate model specification.

*Results for test 1:* The CLM estimated for option 1 had a lower BIC (14892) than the CLM estimated for option 2 (14896) indicating that specifying the model with two alternative-specific constants did not add sufficient explanatory power for inclusion in the model. This result, that one constant provided a better fit than two constants, indicates that there was no tendency for participants to choose the left or right alternative regardless of the attribute levels.

Test 2: Testing for non-linearity in the cost attribute

*Objective of test 2:* To test for evidence of non-linearity in the preferences for the cost attribute.

*Method for test 2:* First, a conditional logit model (CLM) was run assuming a linear and continuous specification for the cost attribute. Next, a CLM was run with the cost attribute effects-coded. All coefficients (regardless of statistical significance) of the cost attribute from this model were plotted against their corresponding attribute levels to allow for a visual inspection of the degree of non-linearity in this attribute (see Figure S4.1). If there was evidence of non-linearity, a series of CLM were estimated with different non-linear specifications for the cost attribute: quadratic, piecewise and log. The measures of fit including the log-likelihood, Akaike Information Criterion (AIC) and Bayesian Information Criterion (BIC) were noted for each CLM.

*Results for test 2:* A visual inspection of Figure S4.1 revealed that there appeared to be evidence of non-linearity in the cost attribute with values of 2,000 SEK having a similar negative impact on choice as values of 1,000 SEK. The upward trajectory after 1,000 SEK suggests that participants experienced a greater disutility at 1,000 SEK when compared with 2,000 SEK. Hence, conditional logit models with non-linear specification of the cost attribute were run. The measures of fit for each model are presented in Table S4.1.

Figure S4.1: Non-linearity in the cost attribute

Table S4.1: Measures of fit for conditional logit models with different non-linear specifications of the cost attribute

|  | **Linear** | **Quadratic** | **Piecewise** | **Log** |
| --- | --- | --- | --- | --- |
| Log-Likelihood | -7410 | -7384 | -7386 | -7400 |
| AIC | 14842 | 14792 | 14795 | 14822 |
| BIC | 14931 | 14889 | 14892 | 14911 |

Table S4.1 indicates that the model with a quadratic specification for cost had the lowest BIC, meaning that this model provided the best model fit to the observed non-linear pattern for the cost attribute. However, when this model was used to estimate willingness-to-pay (WTP) values for the other attributes and levels, it became apparent that the WTP values could not be estimated for some combinations of attributes and levels due to the turning point of the model (1,000 SEK). As such, the piecewise specification (specification with the second lowest BIC) with a break at 1,000 SEK was chosen instead. This model provided a similar BIC value while allowing the estimation of WTP values for all combinations of attributes and levels.

Test 3: Selection of the best-fitting model for the observed choice data

*Objective of test 3:* To identify the best fitting model for the observed choice data.

*Method for test 3:* The conditional logit model (CLM) is the ‘workhorse’ econometric model used to analyse choice data collected from a discrete choice experiment. The CLM assumes that respondents have the same preferences and same level of error variance in their responses. Different model types are available that relax the assumptions made by the CLM. One example is the random parameters logit (RPL) models (uncorrelated and correlated) which allow for preference heterogeneity among respondents. The correlated form of the random parameters logit model also allows for heterogeneity in error variance between participants.

In the analysis of the aggregated data, both uncorrelated and correlated RPL models were compared to the CLM using Bayesian Information Criterion (BIC) where a lower BIC indicates a better model fit.

*Results of test 3:* The uncorrelated RPL model was chosen as the final model as it provided a better model fit over the conditional logit (see Table S4.2). The correlated RPL model did not converge, meaning that the model could not converge to a solution where the log-likelihood has a true maximum i.e. the gains in log-likelihood were always larger than a given threshold.

Table S4.2 Model fit for different model types

| **Model type** | **BIC** |
| --- | --- |
| Conditional logit | 14892 |
| Uncorrelated RPL | 10929 |
| Correlated RPL | Did not converge and no solution found |
| ***BIC****, Bayesian Information Criterion;* ***RPL****, Random parameters logit* | |

**Supplementary Appendix S5 – Illustration of reported health status in survey respondents**

*Objective*: To illustrate the distribution of self-reported health status in the population of respondents.

*Method*: The online survey asked respondents to complete the EQ-5D-5L. The EQ-5D is a generic health-related quality of life instrument which measures health over 5 domains: mobility, self-care, ability to take part in usual activities, pain/discomfort, and anxiety/depression. It comes in versions with either 3 levels (3L) of answers or 5 levels (5L) of answers for each domain. In this study, the Swedish version of the EQ-5D-5L was used.

Once a respondent has completed the EQ-5D-5L, their answers to the questionnaire are converted into a cardinal health utility score, which is anchored on zero (equal to being dead) and one (equal to being in perfect health), using weightings obtained from studies of population preferences for health states in the relevant country. These published weightings are called ‘value sets.’ No official value set exists for the Swedish version of the EQ-5D-5L questionnaire. Hence the published scoring algorithm presented by Burström et al (2020) was used [1] to calculate EQ-5D scores which were plotted in Figure S5.1. This study used time trade off and visual analogue scale tasks followed by least squares and generalised linear regression models to produce a value set.

*Results*: The median utility value was 0.9325 with an interquartile range of 0.1016. Figure S5.1 illustrates the distribution of the resulting health utility scores based on the participants’ answers to the EQ-5D 5L questionnaire. It shows that the utility distribution was bimodal with the first peak at 0.9761 and the second peak at 0.9325. This is commonly reported when modelling health utility data [2].

Figure S5.1: Distribution of health utility values

**References**

1. Burström K, Teni FS, Gerdtham UG, Leidl R, Helgesson G, Rolfson O *et al*. Experience-Based Swedish TTO and VAS Value Sets for EQ-5D-5L Health States. *Pharmacoeconomics*. 2020;38(8):839-56.

2. Hernández Alava M, Wailoo AJ, Ara R. Tails from the peak district: adjusted limited dependent variable mixture models of EQ-5D questionnaire health state utility values. *Value Health*. 2012;15(3):550-61.
